# External validation of an artificial intelligence–based model for retinopathy of prematurity screening using Phoenix ICON retinal images

**DOI:** 10.1016/j.jaapos.2025.104696

**Published:** 2025-11-21

**Authors:** Lizanne A. Derks, Y. Selim Tekin, Sjoukje E. Loudon, Johannes R. Vingerling, Aaron S. Coyner, J. Peter Campbell, Angela M. Tjiam

**Affiliations:** a Department of Ophthalmology, Erasmus University Medical Center, Rotterdam, The Netherlands; b Casey Eye Institute, Oregon Health & Science University, Portland, Oregon

## Abstract

**PURPOSE:**

To assess the performance of a RetCam-trained artificial intelligence (AI) algorithm for the autonomous detection of severe retinopathy of prematurity (ROP) using retinal images acquired with the smaller field-of-view Phoenix ICON retinal camera.

**METHODS:**

Retrospective external validation was performed using Phoenix ICON retinal images captured during ROP screening examinations in a Dutch cohort of infants born in 2021. Images of insufficient quality were excluded via automated quality assessment. Model performances for more-than-mild ROP (MTM-ROP)—type 1 or 2 ROP, or any ROP with pre-plus disease—and for type 1 ROP alone, were expressed as area under the precision-recall curve (AUPRC), sensitivity and specificity.

**RESULTS:**

A total of 4,411 images from 66 infants were captured during 419 individual eye examinations, averaging 67 ± 65 images per infant and 10 ± 6 images per eye examination. Sixty examinations (14.3%) had all images excluded in automated quality assessment. When using the best performance between both eyes to assess infant-level performance, AUPRC was 0.911 (95% CI, 0.638–1.000), sensitivity was 82.0% (95% CI, 73.0–89.0) and specificity was 77.0% (95% CI, 68.1–84.4) for MTM-ROP. For type 1 ROP alone, AUPRC was 0.983 (95% CI, 0.964–1.000), sensitivity was 100.0% (95% CI, 94.7–100.0), and specificity was 72.4% (95% CI, 64.4–79.5).

**CONCLUSIONS:**

The algorithm’s performance with Phoenix ICON is similar to its performance with RetCam. All infants with treatment-requiring type 1 ROP were detected by the algorithm. The presence of eye examinations without images of sufficient quality underlines the need for imaging protocols, especially when using this algorithm, with a smaller field-of-view camera.

Screening plays a crucial role in the timely detection and treatment of retinopathy of prematurity (ROP).^1^ It presents several challenges, though, that can deter ophthalmologists from taking it on as a clinical task, including the time-consuming nature of screening, medicolegal liability claims, training requirements, and logistical issues.^2–4^ Consequently, there can be a disparity between the size of the at-risk population and the number of available ophthalmologists who specialize in ROP, especially in low- and middle-income countries with increasing neonatal survival but limited medical resources.^5^

In recent years, multiple examples of screening tools for diabetic retinopathy based on artificial intelligence (AI) have been approved for clinical use.^6,7^ Similarly, AI could be used in ROP screening to reduce ophthalmologists’ workload through autonomous disease detection and improve screening accessibility via telemedicine in resource scarce regions. The Imaging & Informatics in ROP (i-ROP) research consortium’s deep learning (i-ROP DL) algorithm generates a quantitative vascular severity score (VSS) that has been shown to detect cases of type 1 ROP and more-than-mild ROP (MTM-ROP)—defined as type 1 ROP, type 2 ROP, or any ROP with pre-plus disease—with 100% and 80% sensitivity, respectively.^8^

Prior research has suggested that diagnostic performance of an image-based AI algorithm varies according to the type of camera by which the images are captured.^9,10^ During initial training and subsequent external validation, the i-ROP DL algorithm has been applied to retinal images captured by the RetCam (Natus, Pleasanton, CA) and the Forus 3Nethra (Forus, Bengaluru, Karnataka, India) digital camera systems, with comparable results.^11–14^ Another camera system on the market for ROP screening is the Phoenix ICON (NeoLight, Scottsdale, AZ). Notably, the Phoenix ICON has a reduced field of view (FOV) of 100°; the FOVs of the other two camera systems were 120°–130°. In this study, we report the first validation of the i-ROP DL algorithm for retinal images captured with the Phoenix ICON digital camera system.

## Materials and Methods

This study involved a site in the Netherlands conducting external validation, Erasmus University Medical Center, Rotterdam (“validation site”), and a US site managing the algorithm, Oregon Health & Science University, Portland, Oregon (“model site”). Approval was granted by the Erasmus University Medical Center Institutional Review Board, and the study adhered to the tenets of the Declaration of Helsinki. A data transfer agreement between sites ensured compliance with applicable regulations. Parental informed consent was obtained for all participants, including explicit consent for transferring anonymized retinal images outside the European Union. No financial incentives to participate were provided.

### Study Design and Participants

This retrospective cohort study included preterm infants born in 2021 who were screened for ROP with the Phoenix ICON camera at the validation site by onsite ophthalmologists. Infants were excluded when retinal images taken before ROP treatment or complete regression of ROP were unavailable at the validation site because of prior admission to another hospital.

### Image Selection and Model Output

All retinal images from included infants’ routine ROP screening examinations were extracted at the validation site. Images that failed to capture the retina (e.g., accidentally captured the floor, blankets, infant’s eyelid etc.) and images from post-treatment examinations were manually excluded. The remaining images were passed through the i-ROP-DL algorithm, which supplied an automated quality assessment and a VSS ranging from 1 to 9 for each image. The automated quality assessment algorithm assessed the ability to detect an optic disk in an image, ensuring that the FOV was correct and the image was clear and in focus.^8^ The VSS determined whether referral to in-person screening by an ophthalmologist would have occurred, assuming autonomous model-based screening had been applied to the study cohort.

### Reference Standard Diagnosis

At the validation site, screening duties were shared by two ophthalmologists, and screening was performed according to the then effective national guidelines (Table S1 in Supplement 1, available at jaapos.org). Based on the ophthalmologists’ clinical findings during in-person screening with the Phoenix ICON camera, each eye received an eye-level reference standard diagnosis (RSD) according to the International Classification of ROP (ICROP2 2005).^15^ RSD included ROP stage (no ROP, stage 1–5), zone (zone 1–3, complete vascularization), and the presence of (pre-)plus disease (plus, pre-plus, or no plus). Because the interpretation of plus disease can vary between experts,^16,17^ examinations with a (pre-)plus disease diagnosis or any other descriptions of central vessel abnormalities were reassessed by three different graders using the updated definitions of plus disease and pre-plus disease from the ICROP3 (2021).^18^ The majority diagnosis from the three graders was used. If a majority could not be formed, consensus was reached after joint review.

### Data Set Preparation

Demographics included sex, birthweight (BW), gestational age (GA), postmenstrual age (PMA) at first screening, number of screening examinations, number of available retinal images, and ROP treatment. For each eye, the RSD was further categorized as type 1 or type 2 according to the ETROP criteria,^19^ or as mild ROP or no ROP. In accordance with prior research, we defined MTM-ROP as ETROP type 1 and 2 ROP and cases with pre-plus disease.^8^ RSD data were matched to the VSS and quality assessment data. If multiple images of screened eyes passed the quality assessment, the mean of the VSS values of those images was used as the eye-level VSS.

We also performed infant-level analyses to reflect actual clinical use of the algorithm, where decisions based on model output apply to the child as a whole as opposed to the individual eyes. Infant-level RSD was based on the most severe diagnosis between the two eyes, determined first by the presence of plus disease, second by the highest ROP stage and third by the most posterior zone. For infant-level VSS, the greater value of the two eye-level values was used.

If no images of sufficient quality were available, eye examinations were automatically labeled for referral to an ophthalmologist. The same was done for infant-level analysis when one or both eyes had no images of sufficient quality, or when only one eye was examined during screening.

### Statistical Analysis

Demographics were compared between treated and nontreated infants using the independent samples *t* test or the Fisher’s exact test. The mean VSS and standard deviation were calculated for the ROP stages, zones, (pre-)plus disease and ROP types, and Brown-Forsythe or Mann-Whitney *U* tests were used to compare groups. Eye-level and infant-level model performances for type 1 ROP and MTM-ROP were assessed by calculating the area under the receiver operating curve (AUROC) and precision recall curve (AUPRC), sensitivity, specificity, positive predictive value (PPV) and negative predicting value (NPV) at a referral threshold of VSS = 2.9. The 2.9 threshold was selected for this study to ensure relevance for future clinical implementation, because it is the value currently under evaluation for commercial approval of the model on the RetCam. AUPRC was calculated because severe forms of ROP are generally less common than mild forms of ROP, and an imbalance between the number of positive and negative cases could cause AUROC to be too optimistic. When evaluating real-world infant-level use of the model, we assumed that once an infant was correctly referred by the model, all subsequent monitoring would be provided by an ophthalmologist. The Clopper-Pearson exact method was used to calculate 95% confidence intervals. Statistical significance was assumed at *P* < 0.05. Statistical analysis was performed using SPSS 28 (IBM Corp, Armonk, NY) and Python.

## Results

### Participants

A total of 78 eligible infants were found, of which 66 were included after parental consent. Demographics of treated and nontreated infants are provided in Table 1. GA, BW, and PMA at first screening were lower for treated infants; however, the percentage of girls was higher. Treated infants underwent more screening examinations and had more available retinal images per infant.

### Retinal Images and Quality Assessment

Included infants underwent a total of 213 screening examinations, consisting of 206 bilateral and seven unilateral examinations, totaling 419 eye-level examinations at which 4,410 Phoenix ICON retinal images were taken. On average there were 67 ± 65 images per child, 34 ± 33 images per eye and 10 ± 6 images per screening per eye. A total of 1,223 images passed the quality assessment and were selected for analysis. Rejected images could be placed in four categories: (1) only captured the peripheral retina without the optic nerve; (2) not bright enough; (3) too bright; (4) defocused or blurred due to movement (Supplement 2, available at jaapos.org, Figure S1). After image selection, 60 of 419 (14.3%) eye-level examinations had no quality images, and 51 of 213 (23.9%) infant-level examinations had no quality images of at least one eye or only one eye was screened.

### Reference Standard Diagnosis and VSS

Table 2 shows frequencies of RSD categories and corresponding mean VSS values for all eye-level examinations with at least one image of acceptable quality (n = 359). There were significant differences in mean VSS between the subcategories of each ROP category (*P* <0.001). There were no cases of ROP stage 4 or ROP stage 5 in this dataset. To determine the presence of (pre-)plus disease, 105 out of 359 (29.2%) eye-level examinations were reassessed. The distribution of VSS values for examinations with type 1 ROP versus all other examinations (type 2 ROP, mild ROP and no ROP) is shown in Figure S2 (Supplement 2). Mean VSS was higher for eyes with type 1 ROP compared with eyes without type 1 ROP (*P* < 0.001), with a mean difference in score of 3.9 (95% CI, 3.1–4.7). As seen in Table 2, in eyes with MTM-ROP, mean VSS was higher than in eyes without MTM-ROP (*P* < 0.001), with a mean difference in score of 2.2 (95% CI, 1.7–2.7).

### Eye-level Model Performance

Eye-level AUROC, AUPRC, sensitivity, specificity, PPV, and NPV for type 1 ROP and MTM-ROP are provided in Table 3. Corresponding receiver operating characteristic curves (ROCs) and precision recall curves (PRCs) are shown in Figure S3 (Supplement 2). Notably, the AUPRC is similar to the AUROC for both classifiers, suggesting that model performance is robust even though the dataset is imbalanced. Sixty eye examinations were automatically labeled for referral because of missing quality images; of these, 10 showed MTM-ROP, and 3 showed type 1 ROP.

### Infant-level Model Performance

**FIG 1.**
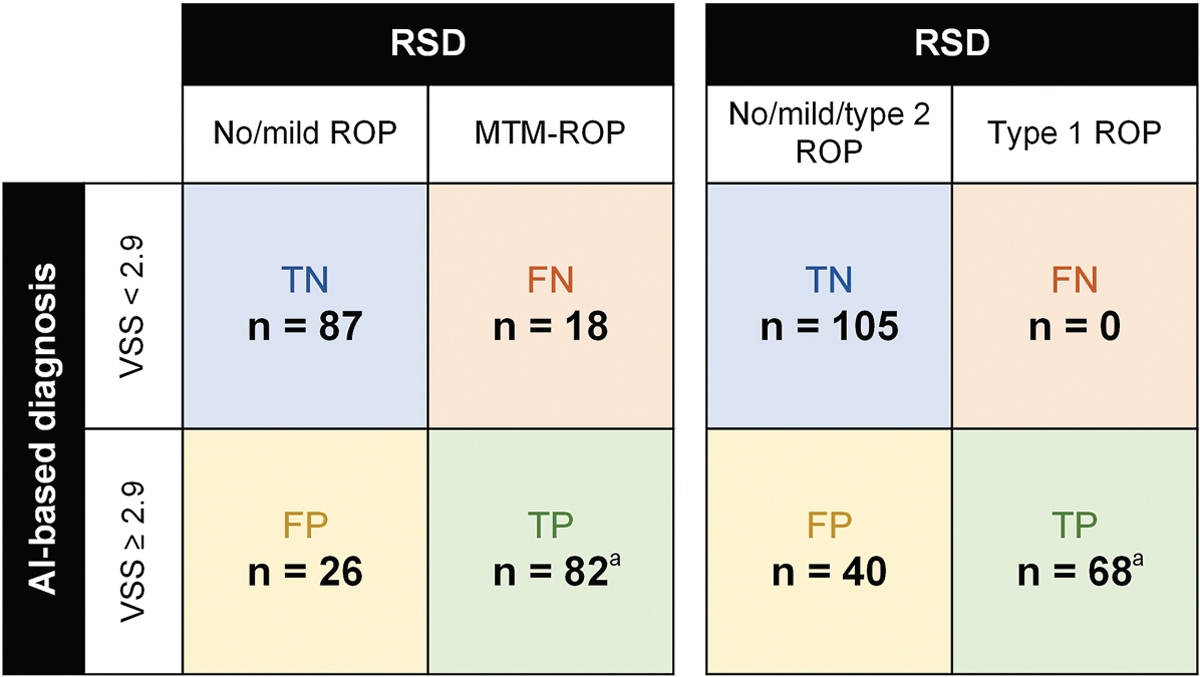
Confusion matrices for MTM-ROP and type 1 ROP at infant level. The confusion matrices show the number of correct and incorrect allocations by the i-ROP DL algorithm (AI-based diagnosis) when using the diagnosis by the ophthalmologist as a reference (RSD). ^a^Includes n = 51 automatic referrals (no quality images available). *FN*, false negative; *FP*, false positive; *MTM*, more than mild; *ROP*, retinopathy of prematurity; *RSD*, reference standard diagnosis; *TN*, true negative; *TP*, true positive; *VSS*, vascular severity score.

Infant-level AUROC, AUPRC, sensitivity, specificity, PPV, and NPV for type 1 ROP and MTM-ROP are provided in Table 3. Corresponding ROCs and PRCs are shown in Figure S4 (Supplement 2). Figure 1 shows the confusion matrices with true-positives, false-positives, true-negatives, and false-negatives for MTM-ROP and type 1 ROP at infant level. A total of 51 examinations were automatically labeled for referral because of missing quality images or because only one eye was examined, of which 10 showed MTM-ROP, and 4 showed type 1 ROP. No cases of type 1 ROP were missed in infant-level analysis. One infant that received ROP treatment had not been diagnosed with type 1 ROP. The algorithm referred this infant prior to treatment, albeit due to missing quality images.

## Discussion

This study is the first external validation of the RetCam-trained i-ROP DL algorithm using images from the Phoenix ICON retinal camera. Although the Phoenix ICON has a narrower FOV relative to RetCam (100° vs 120°−130°), the i-ROP DL algorithm demonstrated high performance in detecting MTM-ROP and type 1 ROP, with AUPRC values of 0.911 and 0.983, respectively. At a VSS cut-off of 2.9, the algorithm achieved 100% sensitivity for type 1 ROP detection, indicating that no infants with treatment-requiring ROP would be missed. These results imply that this AI-based screening tool is effective when the smaller FOV of Phoenix ICON images are used, despite its training on larger FOV images.

Our reported infant-level sensitivity of 100% for type 1 ROP aligns with prior studies. In US and Indian cohorts, sensitivity was 100.0% for RetCam images and 97.3% for 3Nethra Forus images at a VSS cut-off of 3.1.^8^ In a separate North American study with RetCam images, a sensitivity of 94.0% was found at a VSS cut-off of 3.0.^11^ Together with this current study, the i-ROP DL algorithm has demonstrated high sensitivity across different datasets with varying patient demographics and camera systems. High sensitivity is crucial for the clinical applicability of this algorithm, because missing a case of type 1 comes with a consequent risk of lifelong visual impairment. However, to expand the impact on clinical practice, the algorithm’s specificity could be optimized to further reduce workload for ophthalmologists. In our cohort, infant-level specificity was 72.4% for type 1 ROP. Because model sensitivity and specificity are a direct result of the chosen cut-off value, further research could identify the optimal, possibly camera-specific, cut-off value that produces 100% sensitivity and the highest possible specificity.

Not all cases of MTM-ROP were referred by the algorithm. Type 1 ROP is typically the only form of ROP requiring treatment to prevent vision loss. Thus, achieving 100% sensitivity for the detection of MTM-ROP may not be critical to the usability of the algorithm. Yet, one infant in our cohort was treated prior to developing type 1 ROP. This infant was referred by the model due to poor image quality, and it is unclear whether the algorithm would have detected the case with better images. Several studies have reported on non–type 1 ROP treatment, with common reasoning for treatment being pre-plus disease, structural retinal changes, persistent ROP at advanced PMA, persistent avascular retina, and logistical considerations.^20–23^ Therefore, when applying autonomous AI-driven screening to ROP, safeguards that allow for ophthalmologists’ clinical judgement in cases of non–type 1 ROP that might benefit from treatment could be warranted.

There are limitations to this study. First, the number of retinal images rejected during quality assessment was relatively high, which might have slightly distorted model performance because examinations without quality images were automatically labeled for referral. The i-ROP DL algorithm was originally trained on images from five standard FOVs (superior, inferior, nasal, temporal, and posterior), a protocol not implemented at the validation site. The Phoenix ICON’s smaller FOV limits optic nerve visibility in peripheral retinal images, reducing the number of images with the potential to be read by the algorithm. Moreover, pigmentation differences in the diverse patient population at the validation site, combined with camera settings (ie, gain and intensity), may have led to images being too dark or too bright for analysis. A standardized image acquisition protocol focusing on optic nerve visibility, such as the protocol described by the e-ROP study,^24^ could reduce image rejection rates. Second, we performed this study with data from a cohort that is substantially smaller than in prior validation studies for this algorithm. Even though we found similar model performance for Phoenix ICON images compared to prior studies with different camera systems, prospective validation with a larger dataset is needed.

In conclusion, this external validation study demonstrates that the i-ROP DL algorithm, applied for the first time to Phoenix ICON images, has similar performance compared to the wider FOV images it was originally trained on. Autonomous screening for ROP has the potential to reduce workload for ophthalmologists^8^; however, implementation requires investments in imaging acquisition training for non-ophthalmologists and expensive camera equipment. Current low-cost camera systems for ROP screening, including smartphone-based devices that only require the addition of a 20 D, 28 D or 40 D lens, can capture a FOV up to 90°.^25^ The finding that the i-ROP DL algorithm can be applied to a smaller FOV could pave the way to further validation of this technology, preferably with standardized image acquisition protocols, for lower-cost alternatives such as smartphones. This may have important implications for the future of ophthalmological care for preterm infants worldwide, especially in settings where medical resources and trained ophthalmologists are scarce.

## Supplementary Material

1

2

## Figures and Tables

**Table 1.** Patient demographics treated versus non-treated infants^a^

Patient characteristics	Total (N = 66)	Non-treated infants (n = 54)	Treated infants (n = 12)	Mean difference (95% CI)	*P* value

Female sex	30 (45.5)	21 (38.9)	9 (75.0)	NA	0.029
Gestational age, weeks	27.4 ± 2.4	27.9 ± 2.3	25.2 ± 1.1	2.7 (1.8 to 3.6)	<0.001
Birthweight, g	963.5 ± 307.5	1026.1 ± 299.3	682.1 ± 147.5	344.0 (166.0 to 521.9)	<0.001
PMA first screening, weeks	33.3 ± 2.2	33.6 ± 2.3	31.6 ± 0.7	2.0 (1.3 to 2.7)	<0.001
Number of screenings	3.4 ± 3.0	2.9 ± 2.7	5.9 ± 2.9	−3.0 (−5.0 to −1.1)	<0.001
Number of available images	66.8 ± 65.4	52.9 ± 56.3	129.7 ± 68.9	−76.8 (−114.2 to −39.4)	<0.001

*NA*, not applicable; *PMA*, postmenstrual age.

a Demographic data of included infants, with a separate analysis of demographical data for infants that were and infants that were not treated for ROP. Female sex presented as number (%), all other variables presented as mean and standard deviation. Differences between groups were assessed using the Fisher’s exact test for categorical variables and using the independent sample *t* test for numerical variables.

**Table 2.** Eye-level frequencies and mean VSS per RSD category^a^

RSD category	No. examinations (%), no. = 359	VSS, mean ± SD	*P* value

ROP stage			
No ROP	111 (30.9)	1.6 ± 0.7	<0.001
Stage 1	87 (24.2)	2.1 ± 1.3	
Stage 2	111 (30.9)	2.7 ± 1.6	
Stage 3	50 (13.9)	4.4 ± 2.2	
Stage 4	0	NA	
Stage 5	0	NA	
ROP zone			
Complete vascularization	36 (10.0)	1.8 ± 0.9	<0.001
Zone 3	28 (7.8)	1.8 ± 1.1	
Zone 2	281 (78.3)	2.5 ± 1.7	
Zone 1	14 (3.9)	4.3 ± 2.5	
Presence of plus disease			
No plus	287 (79.9)	2.0 ± 1.0	<0.001
Pre-plus	43 (12.0)	3.6 ± 1.9	
Plus	29 (8.1)	5.9 ± 2.0	
ETROP-type			
No ROP	111 (30.9)	1.6 ± 0.7	<0.001
Mild ROP	183 (51.0)	2.3 ± 1.2	
Type 2 ROP	39 (10.9)	3.5 ± 1.9	
Type 1 ROP	26 (7.2)	6.1 ± 2.0	
MTM-ROP			
No/Mild ROP	273 (76.0)	1.9 ± 1.0	<0.001
MTM-ROP	86 (24.0)	4.1 ± 2.3	

*ETROP*, Early Treatment of ROP Study; *MTM-ROP*, more than mild ROP; *NA*, not applicable; *ROP*, retinopathy of prematurity; *RSD*, reference standard diagnosis; VSS, vascular severity score.

a Frequencies and mean vascular severity score per diagnostic ROP category calculated using individual eye examinations (eye-level). Mean VSS values between categories were compared using the Brown-Forsythe test or Mann-Whitney *U* test.

**Table 3.** Eye-level and infant-level detection of type 1 ROP and MTM-ROP using cut-off VSS = 2.9^a^

Classifier	AUROC (95% CI)	AUPRC (95% CI)	Sensitivity % (95% CI)	Specificity % (95% CI)	PPV % (95% CI)	NPV % (95% CI)

Eye-level						
Type 1 ROP	0.977 (0.956–0.997)	0.957 (0.931–0.984)	96.5 (90.1–99.3)	79.3 (74.5–83.5)	54.6 (46.3–62.6)	98.9 (96.8–99.8)
MTM ROP	0.878 (0.838–0.917)	0.869 (0.607–1.000)	76.7 (69.0–83.3)	85.3 (80.6–89.3)	73.7 (65.9–80.5)	87.3 (82.7–91.0)
Infant-level						
Type 1 ROP	0.990 (0.980–1.000)	0.983 (0.964–1.000)	100.0 (94.7–100.0)	72.4 (64.4–79.5)	63.0 (53.1–72.1)	100.0 (96.5–100.0)
MTM-ROP	0.886 (0.838–0.934)	0.911 (0.638–1.000)	82.0 (73.0–89.0)	77.0 (68.1–84.4)	75.9 (66.7–83.6)	82.9 (74.3–89.5)

*AUPRC*, area under the precision recall curve; *AUROC*, area under the receiver operating curve; *CI*, confidence interval; *MTM*, more than mild; *NPV*, negative predictive value; *PPV*, positive predictive value; *ROP*, retinopathy of prematurity.

a Model performance for type 1 ROP and MTM-ROP calculated for individual eye examinations (eye-level) and for combined examination of both eyes (infant-level).

## References

[1] Reynolds JD, Dobson V, Quinn GE, , CRYO-ROP and LIGHT-ROP Cooperative Study Groups. Evidence-based screening criteria for retinopathy of prematurity: natural history data from the CRYO-ROP and LIGHT-ROP studies. Arch Ophthalmol 2002;120:1470–76. 12427059 10.1001/archopht.120.11.1470

[2] Kemper AR, Freedman SF, Wallace DK. Retinopathy of prematurity care: patterns of care and workforce analysis. J AAPOS 2008;12:344–8. 18440256 10.1016/j.jaapos.2008.02.012 PMC2566307

[3] Kemper AR, Wallace DK. Neonatologists’ practices and experiences in arranging retinopathy of prematurity screening services. Pediatrics 2007;120:527–31. 17766525 10.1542/peds.2007-0378 PMC2132441

[4] Vinekar A, Gangwe A, Agarwal S, Kulkarni S, Azad R. Improving retinopathy of prematurity care: a medico-legal perspective. Asia Pac J Ophthalmol (Phila) 2021;10:437–41. 34456232 10.1097/APO.0000000000000388

[5] Bowe T, Nyamai L, Ademola-Popoola D, The current state of retinopathy of prematurity in India, Kenya, Mexico, Nigeria, Philippines, Romania, Thailand, and Venezuela. Digit J Ophthalmol 2019;25:49–58. 32076388 10.5693/djo.01.2019.08.002 PMC7001648

[6] US Food & Drug Administration Artificial Intelligence and Machine Learning (AI/ML)-Enabled Medical Devices, https://www.fda.gov/medical-devices/software-medical-device-samd/artificial-intelligence-and-machine-learning-aiml-enabled-medical-devices. Accessed January 5, 2025.

[7] Hutton D Eyenuk gets greenlight to market AI screening system in European Union, https://www.ophthalmologytimes.com/view/eyenuk-gets-greenlight-to-market-ai-screening-system-in-european-union. Accessed January 5, 2025.

[8] Coyner AS, Murckan T, Oh MA, Multinational external validation of autonomous retinopathy of prematurity screening. JAMA Ophthalmol 2024;142:327–35. 38451496 10.1001/jamaophthalmol.2024.0045 PMC10921347

[9] Srinivasan R, Surya J, Ruamviboonsuk P, Chotcomwongse P, Raman R. Influence of different types of retinal cameras on the performance of deep learning algorithms in diabetic retinopathy screening. Life (Basel) 2022;12:1610. 36295045 10.3390/life12101610 PMC9604597

[10] Lim G, Bellemo V, Xie Y, Lee XQ, Yip MYT, Ting DSW. Different fundus imaging modalities and technical factors in AI screening for diabetic retinopathy: a review. Eye Vis (Lond) 2020;7:21. 32313813 10.1186/s40662-020-00182-7 PMC7155252

[11] Redd TK, Campbell JP, Brown JM, Imaging and Informatics in Retinopathy of Prematurity (i-ROP) Research Consortium. Evaluation of a deep learning image assessment system for detecting severe retinopathy of prematurity. Br J Ophthalmol 2018. bjophthalmol-2018–313156. 10.1136/bjophthalmol-2018-313156 PMC7880608 30470715

[12] Brown JM, Campbell JP, Beers A, Automated diagnosis of plus disease in retinopathy of prematurity using deep convolutional neural networks. JAMA Ophthalmol 2018;136:803–10. 29801159 10.1001/jamaophthalmol.2018.1934 PMC6136045

[13] Taylor S, Brown JM, Gupta K, Monitoring disease progression with a quantitative severity scale for retinopathy of prematurity using deep learning. JAMA Ophthalmol 2019;137:1022–8. 31268518 10.1001/jamaophthalmol.2019.2433 PMC6613341

[14] Campbell JP, Kim SJ, Brown JM, Evaluation of a deep learning-derived quantitative retinopathy of prematurity severity scale. Ophthalmology 2021;128:1070–76. 33121959 10.1016/j.ophtha.2020.10.025 PMC8076329

[15] International Committee for the Classification of Retinopathy of Prematurity. The International Classification of Retinopathy of Prematurity revisited. Arch Ophthalmol 2005;123:991–9. 16009843 10.1001/archopht.123.7.991

[16] Campbell JP, Ryan MC, Lore E, Diagnostic discrepancies in retinopathy of prematurity classification. Ophthalmology 2016;123:1795–801. 27238376 10.1016/j.ophtha.2016.04.035 PMC4958515

[17] Campbell JP, Kalpathy-Cramer J, Erdogmus D, Plus disease in retinopathy of prematurity: a continuous spectrum of vascular abnormality as a basis of diagnostic variability. Ophthalmology 2016;123:2338–44. 27591053 10.1016/j.ophtha.2016.07.026 PMC5077639

[18] Chiang MF, Quinn GE, Fielder AR, International Classification of Retinopathy of Prematurity, Third Edition. Ophthalmology 2021;128:e51–68. 34247850 10.1016/j.ophtha.2021.05.031 PMC10979521

[19] Good WV. Early Treatment for Retinopathy of Prematurity Cooperative Group. Final results of the Early Treatment for Retinopathy of Prematurity (ETROP) randomized trial. Trans Am Ophthalmol Soc 2004;102:233–50. 15747762 PMC1280104

[20] Rajan RP, Kohli P, Babu N, Dakshayini C, Tandon M, Ramasamy K. Treatment of retinopathy of prematurity (ROP) outside International Classification of ROP (ICROP) guidelines. Graefes Arch Clin Exp Ophthalmol 2020;258:1205–10. 32322963 10.1007/s00417-020-04706-8

[21] Gupta MP, Chan RVP, Anzures R, Practice patterns in retinopathy of prematurity treatment for disease milder than recommended by guidelines. Am J Ophthalmol 2016;163:1–10. 26705094 10.1016/j.ajo.2015.12.005 PMC4769781

[22] Lemaître D, Barjol A, Abdelmassih Y, Treatment outside the recommended guidelines for retinopathy of prematurity (ROP): prevalence, characteristics, and issues. J Clin Med 2021;11:39. 35011779 10.3390/jcm11010039 PMC8745039

[23] Sanghi G, Gangwe A, Das P. Evidence based management of retinopathy of prematurity: more than meets the eye. Clin Epidemiol Glob Health 2024;26:101530.

[24] Quinn GE, e-ROP Cooperative Group. Telemedicine approaches to evaluating acute-phase retinopathy of prematurity: study design. Ophthalmic Epidemiol 2014;21:256–67. 24955738 10.3109/09286586.2014.926940 PMC4861056

[25] Goyal A, Gopalakrishnan M, Anantharaman G, Chandrashekharan DP, Thachil T, Sharma A. Smartphone guided wide-field imaging for retinopathy of prematurity in neonatal intensive care unit—a Smart ROP (SROP) initiative. Indian J Ophthalmol 2019;67:840–45. 31124499 10.4103/ijo.IJO_1177_18 PMC6552601

