# Supplementary material for: External validation of an artificial intelligence–based model for retinopathy of prematurity screening using Phoenix ICON retinal images
Source: J AAPOS. Author manuscript; Available in PMC 2026 Jul 8. (PMC13345673; doi:10.1016/j.jaapos.2025.104696)
Supplement: 1 [file NIHMS2187166-supplement-1.docx]

Derks LA, Tekin YS, Loudon SE, Vingerling JR, Coyner AS, Campbell JP, Tjiam AM. External validation of an artificial intelligence-based model for retinopathy of prematurity screening using Phoenix ICON retinal images.

J AAPOS online supplement.

Supplement 1. eTable

Table S1. National ROP screening guidelines

| Eligibility for screening | All infants with GA< 30 weeks and/or BW < 1250 g. |
| --- | --- |
|  | All infants with GA 30-32 weeks and/or BW 1250-1500 g and one or more of the following risk factors:   - artificial ventilation; - sepsis; - necrotizing enterocolitis (NEC); - postnatal use of corticosteroids ; - use of cardiotonica to combat hypotension.   Or: all infants GA<32 weeks and/or BW<1500 g when reports of these risk factors are unreliable. |
| First screening | First screening is recommended at 5 weeks (35-42 days) PNA, but not before 31 weeks PMA. |
| Follow-up of screening | Screening at least twice per week in the case of ROP with plus disease. |
|  | Weekly screening in the case of:   1. Avascular retina in zone I; 2. ROP 1-2 in zone I without plus disease; 3. ROP 2-3 in zone II without plus disease; 4. Regressing ROP in zone I; 5. Assessment of the fundus not possible. |
|  | Screening once every two weeks in the case of:   1. Avascular retina in zone II without ROP; 2. ROP 1 in zone II; 3. ROP 1-2 in zone III; 4. Regressing ROP in zone II-III. |
| Reducing screening frequency | The screening frequency can be reduced when:   - The severity of ROP decreases in multiple consecutive screening visits; - The infant reaches 40 weeks PMA without ROP. |
| Ending screening | Screening ends when one of the following criteria is met:   - Complete vascularization of the retina; - Apparent regression of ROP at 40 weeks PMA without plus disease; - Apparent growth of the retinal vessels over the demarcation line toward the ora serrata; - Apparent regression of ROP with the ridge changing from pink to white. |
| BW, birthweight; GA, gestational age; PMA, postmenstrual age; PNA, postnatal age; ROP, retinopathy of prematurity. | |
|  | |
