# Supplementary material for: External validation of an artificial intelligence–based model for retinopathy of prematurity screening using Phoenix ICON retinal images
Source: J AAPOS. Author manuscript; Available in PMC 2026 Jul 8. (PMC13345673; doi:10.1016/j.jaapos.2025.104696)
Supplement: 2 [file NIHMS2187166-supplement-2.pdf]

Derks LA, Tekin YS, Loudon SE, Vingerling JR, Coyner AS, Campbell JP, Tjiam AM. External validation of an artificial intelligence-based model for retinopathy of prematurity screening using Phoenix ICON retinal images. J AAPOS online supplement.

Supplement 2. eFigures

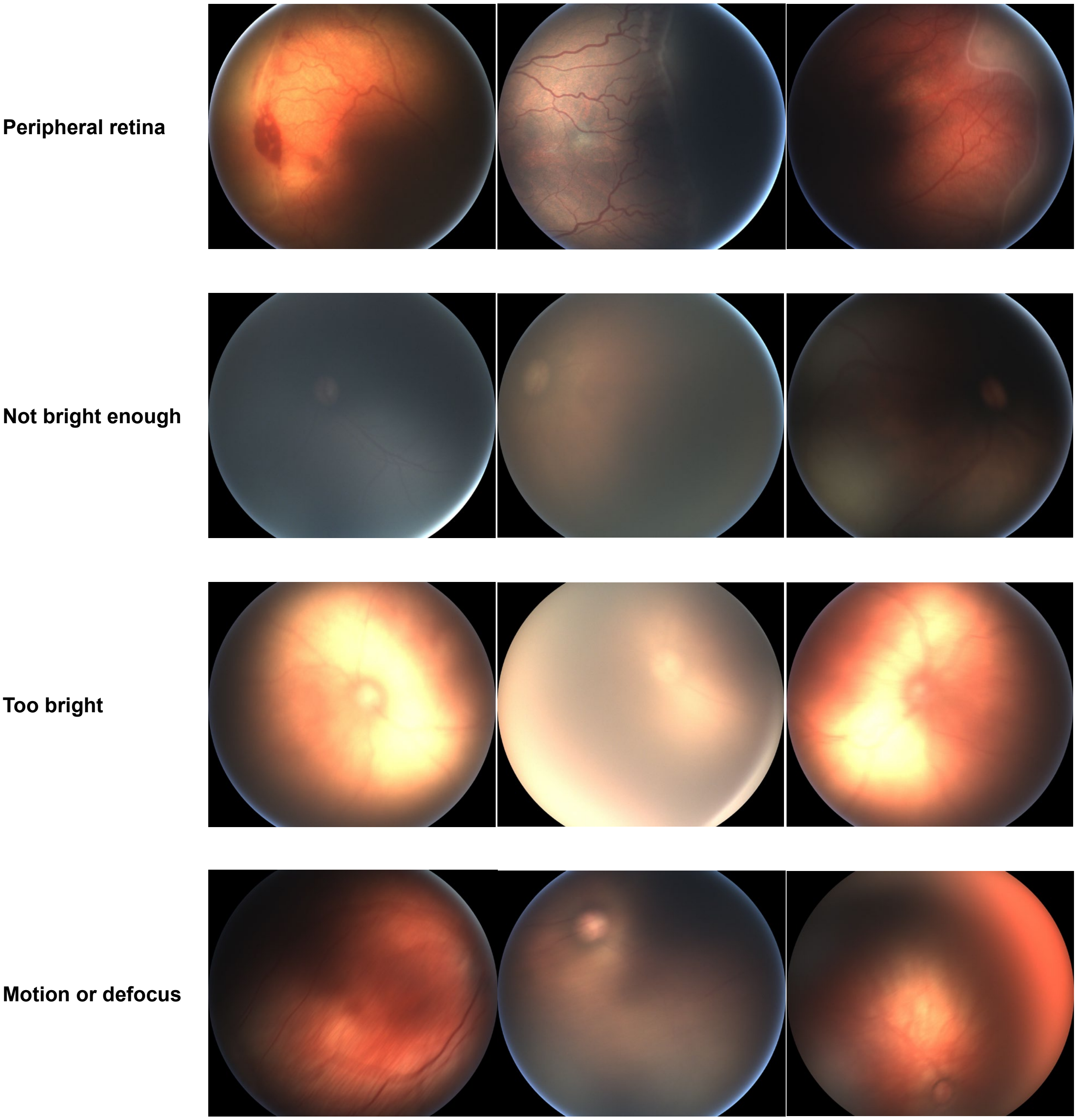

FIG S1. Examples of images that were not selected for analysis during automated quality assessment. Shown are images that did not pass the automated quality assessment, as the algorithm could not identify the optic disk. We identified four reasons for insufficient visibility of the optic disk; the image captured the peripheral retina so the optic disk was outside the field-of-view of the camera, the image was not bright enough, the image was too bright, or the optic disk could not be detected because the image was blurred through motion or defocus.

Copyright © 2025 by the American Association for Pediatric Ophthalmology and Strabismus

Derks LA, Tekin YS, Loudon SE, Vingerling JR, Coyner AS, Campbell JP, Tjiam AM. External validation of an artificial intelligence-based model for retinopathy of prematurity screening using Phoenix ICON retinal images. J AAPOS online supplement.

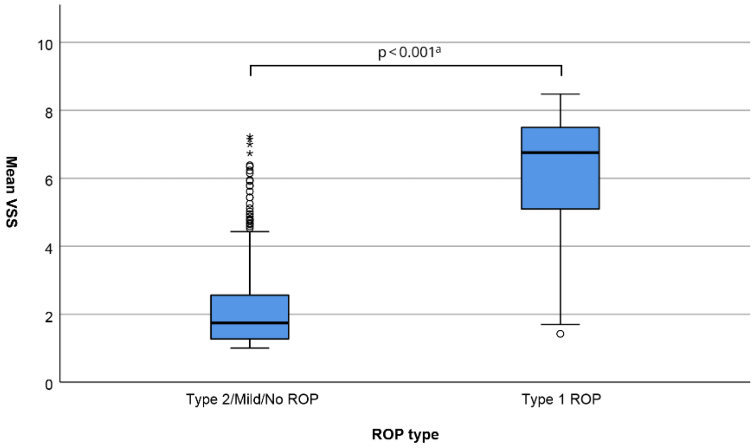

FIG S2. Boxplots of mean VSS by ETROP-type showing the distribution of mean VSS for eye examinations with type 1 ROP versus eye examinations with other forms of ROP or no ROP. [a]Mann-Whitney *U* test. *MTM*, more than mild; *ROP*, retinopathy of prematurity; *VSS*, vascular severity score.

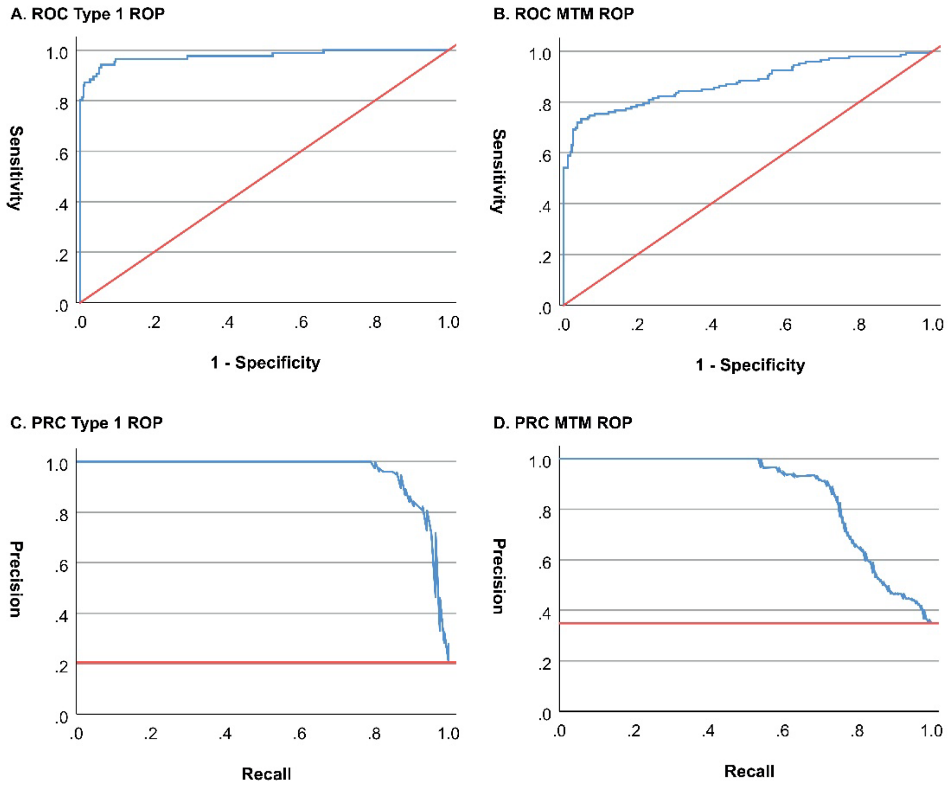

FIG S3. Eye-level ROC and PRC for type 1 ROP and MTM-ROP. A, ROC type 1 ROP. B, ROC MTM-ROP. C, PRC type 1 ROP. D, PRC MTM-ROP. The ROC and PRC (blue) show the overall diagnostic performance of the i-ROP DL algorithm for type 1 ROP and MTM-ROP compared to a no-skill reference line (red). In eye-level analysis, VSS and ROP diagnosis of each eye were used for each eye examination. *MTM*, more than mild; *PRC*, precision-recall curve; *ROC*, receiver operating characteristic curve; *ROP*, retinopathy of prematurity.

Copyright © 2025 by the American Association for Pediatric Ophthalmology and Strabismus

Derks LA, Tekin YS, Loudon SE, Vingerling JR, Coyner AS, Campbell JP, Tjiam AM. External validation of an artificial intelligence-based model for retinopathy of prematurity screening using Phoenix ICON retinal images. J AAPOS online supplement.

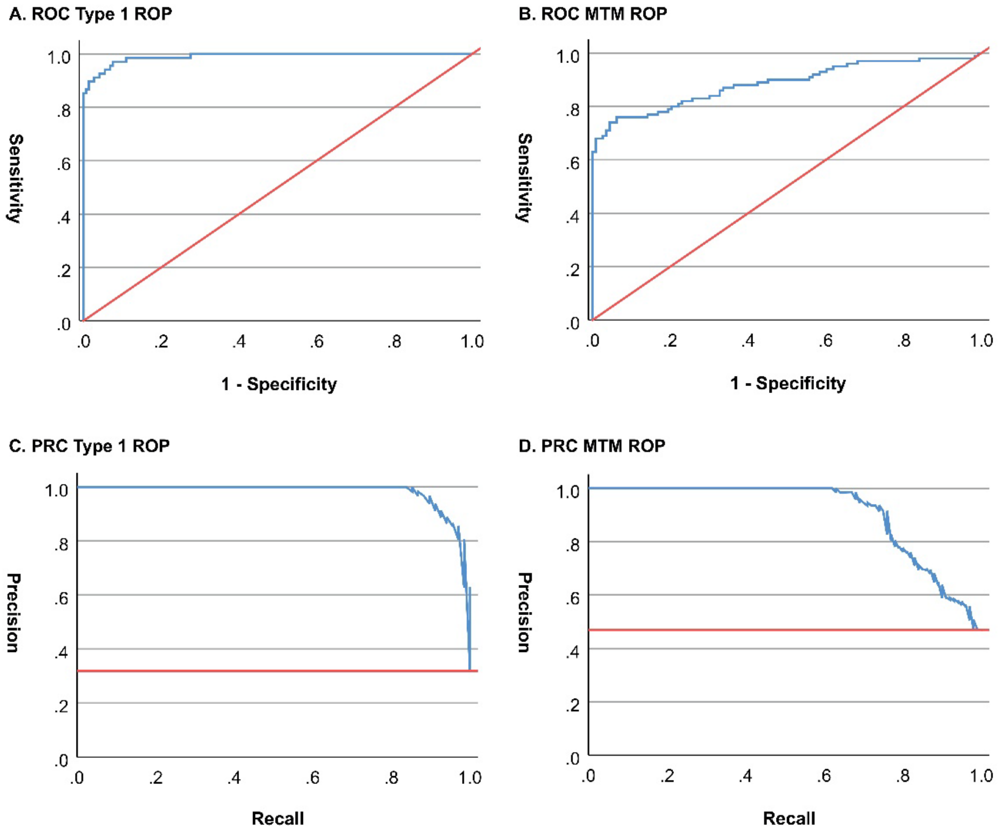

FIG S4. Infant-level ROC and PRC for type 1 ROP and MTM-ROP. A, ROC type 1 ROP. B, ROC MTM-ROP. C, PRC type 1 ROP. D, PRC MTM-ROP. The ROC and PRC (blue) show the overall diagnostic performance of the i-ROP DL algorithm for type 1 ROP and MTM-ROP compared to a no-skill reference line (red). In infant-level analysis, the highest VSS and ROP diagnosis between the two eyes were used for each eye exam. *MTM*, more than mild; *PRC*, precision-recall curve; *ROC*, receiver operating characteristic curve; *ROP*, retinopathy of prematurity.

Copyright © 2025 by the American Association for Pediatric Ophthalmology and Strabismus
